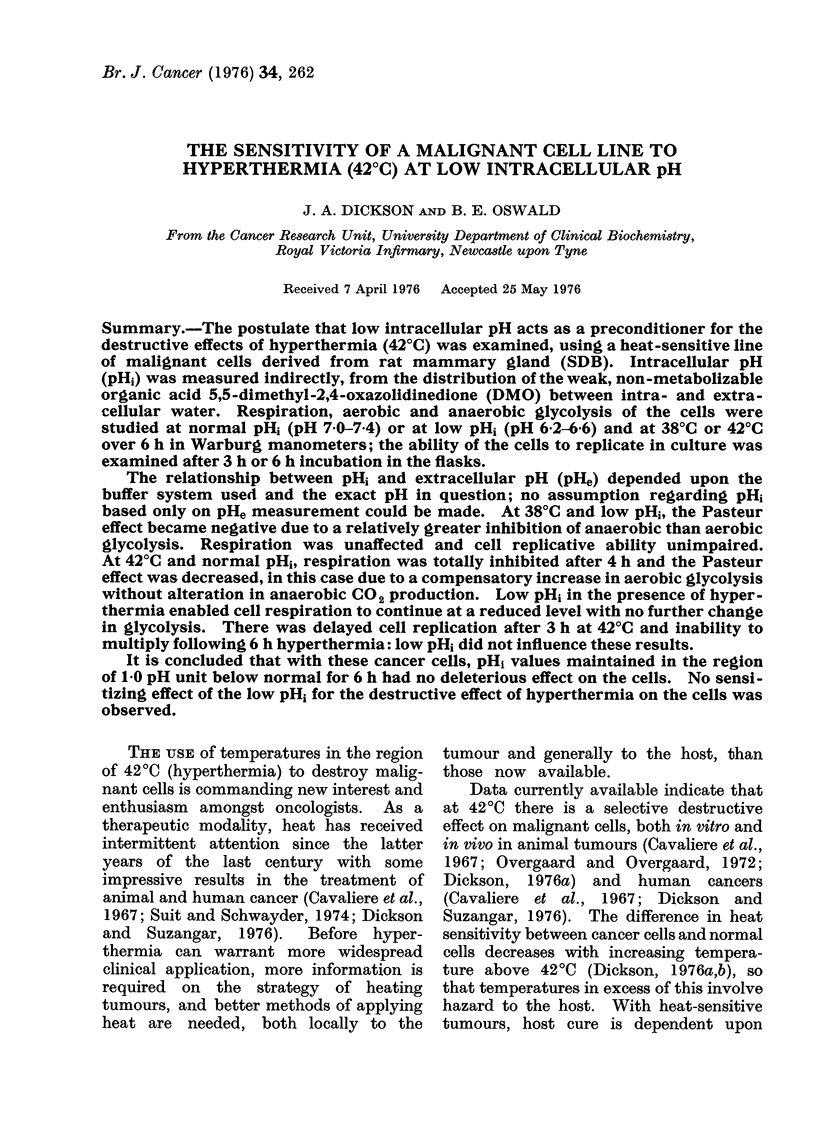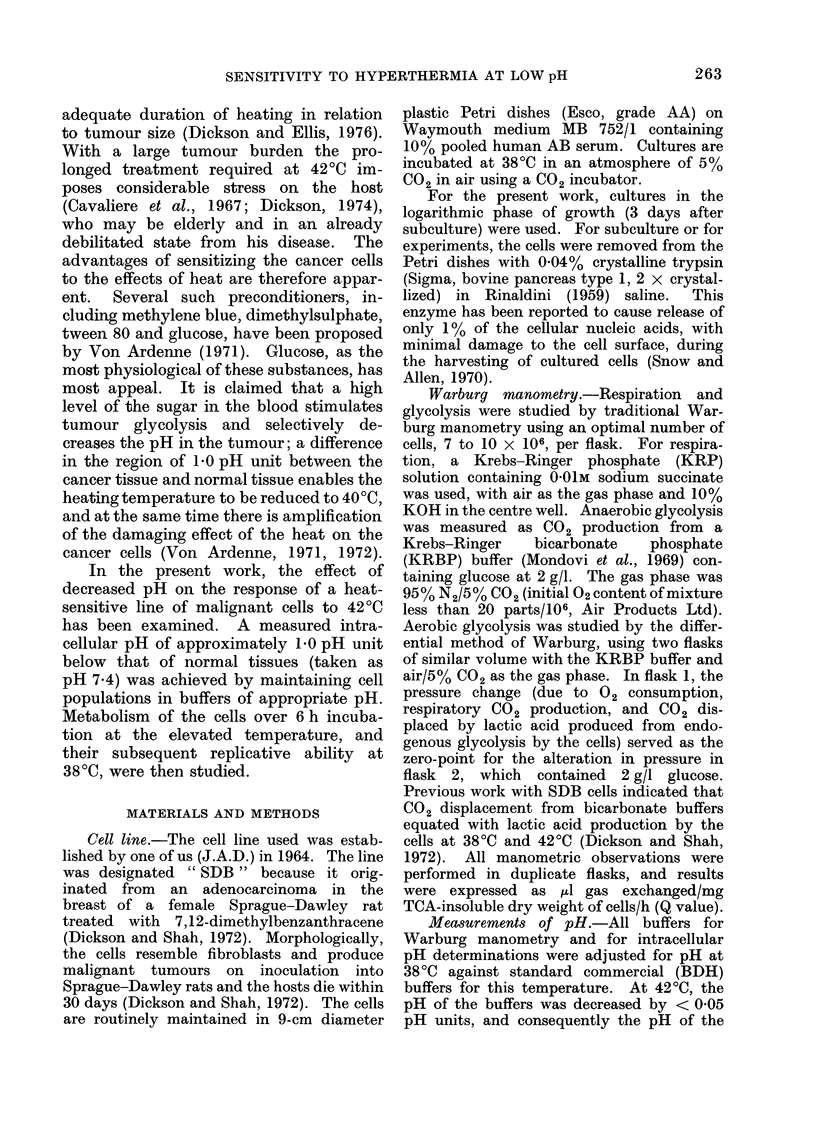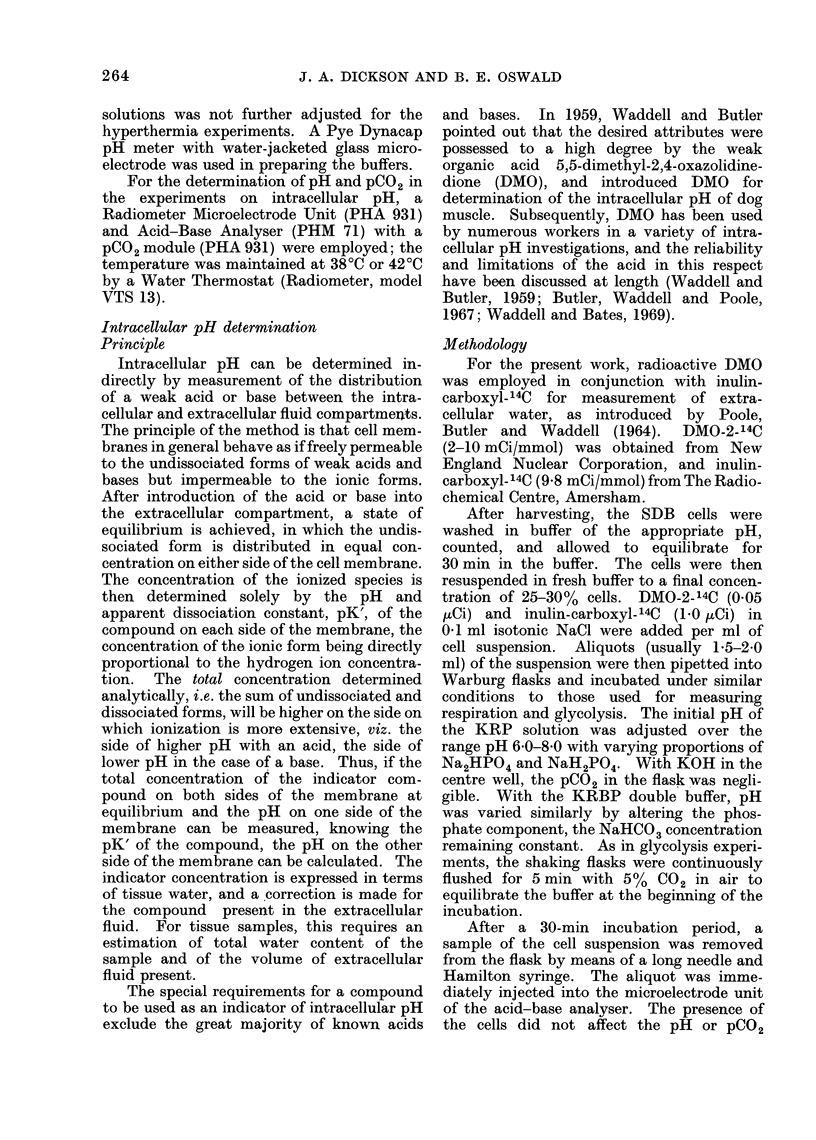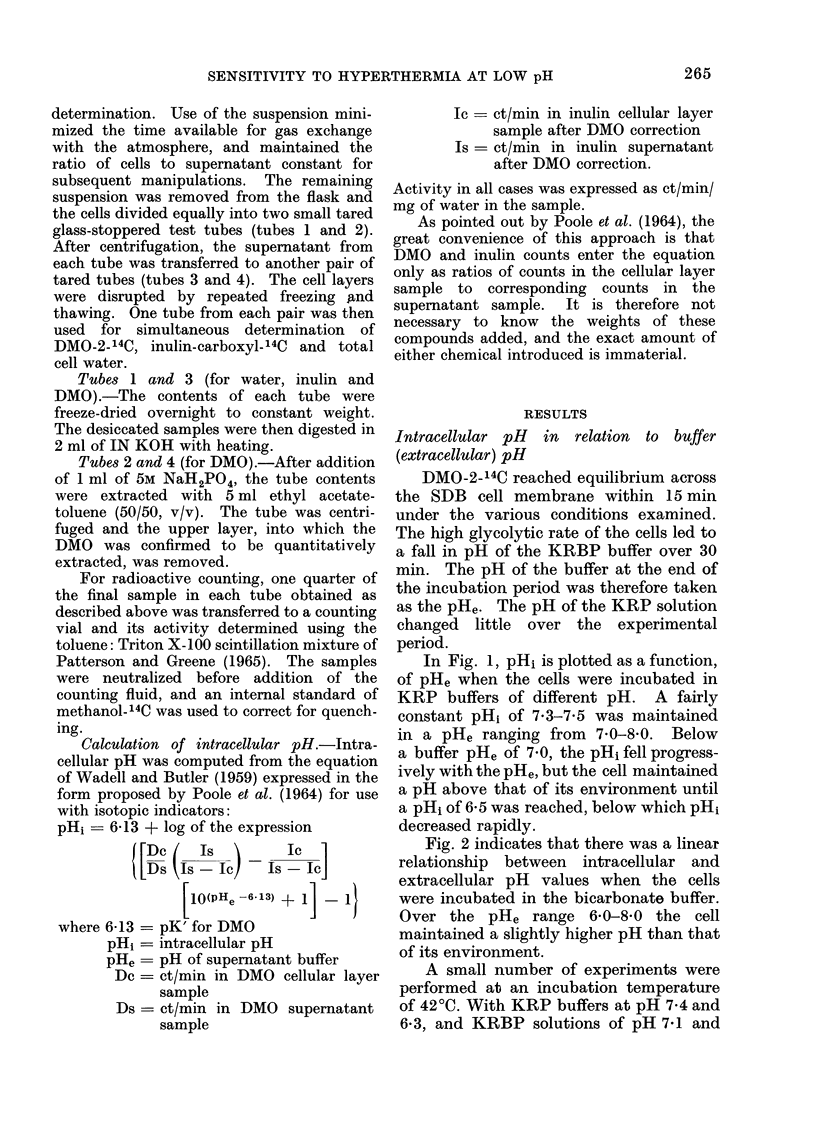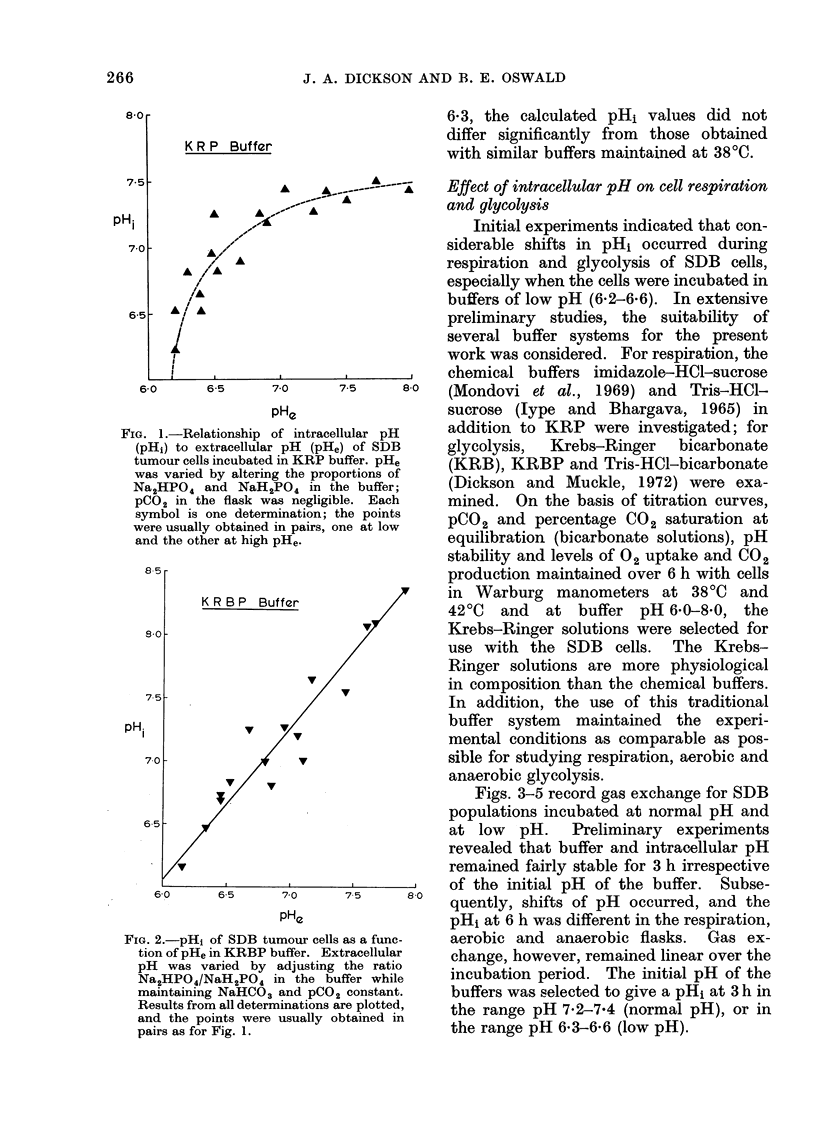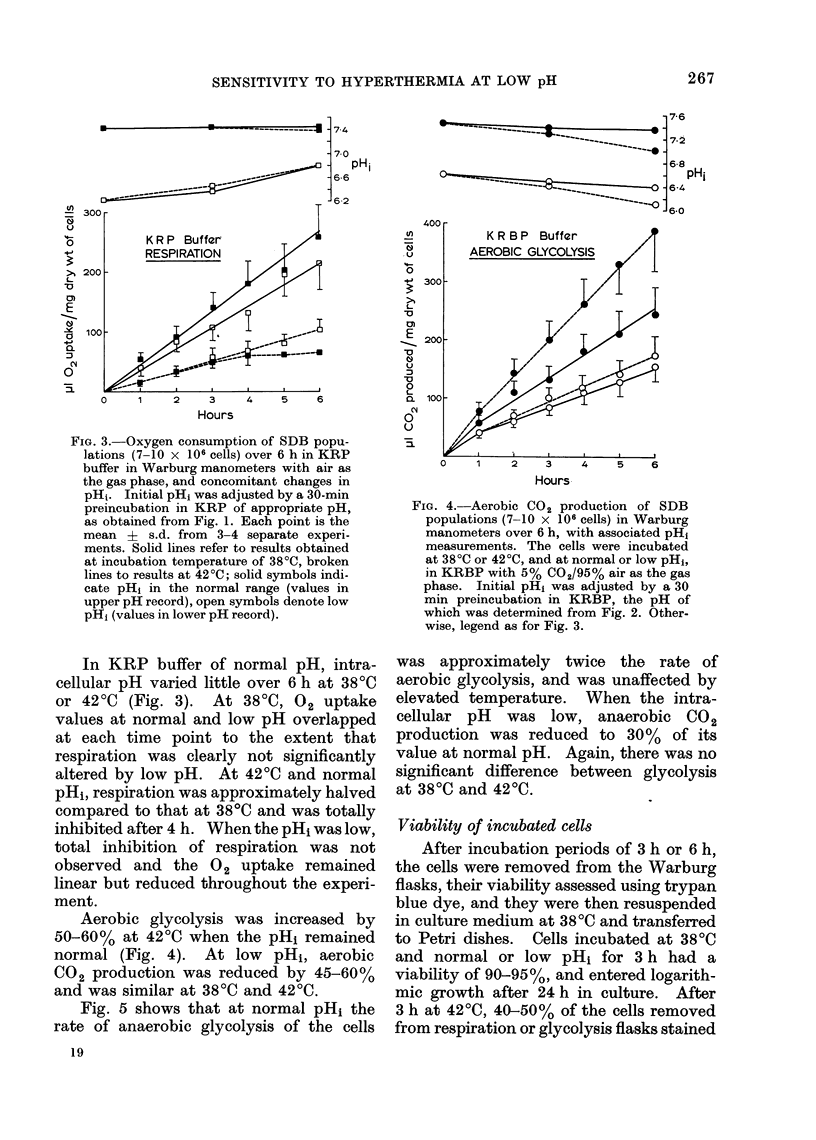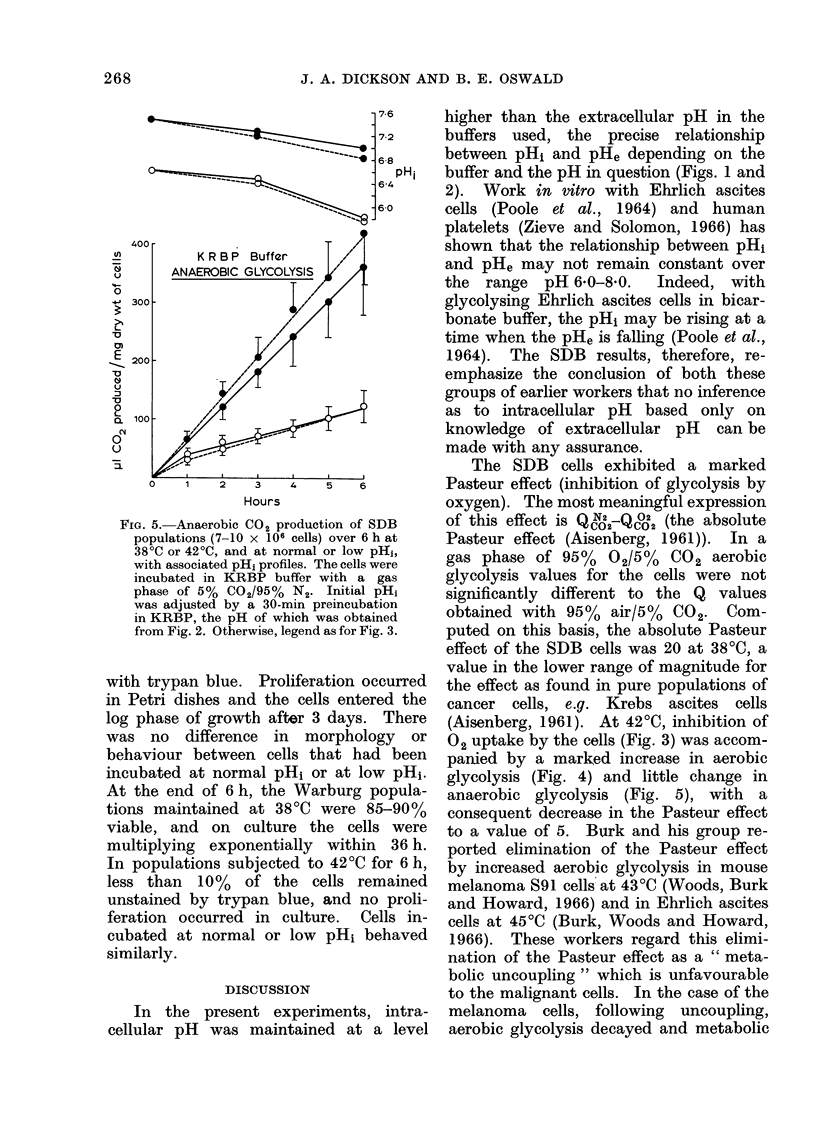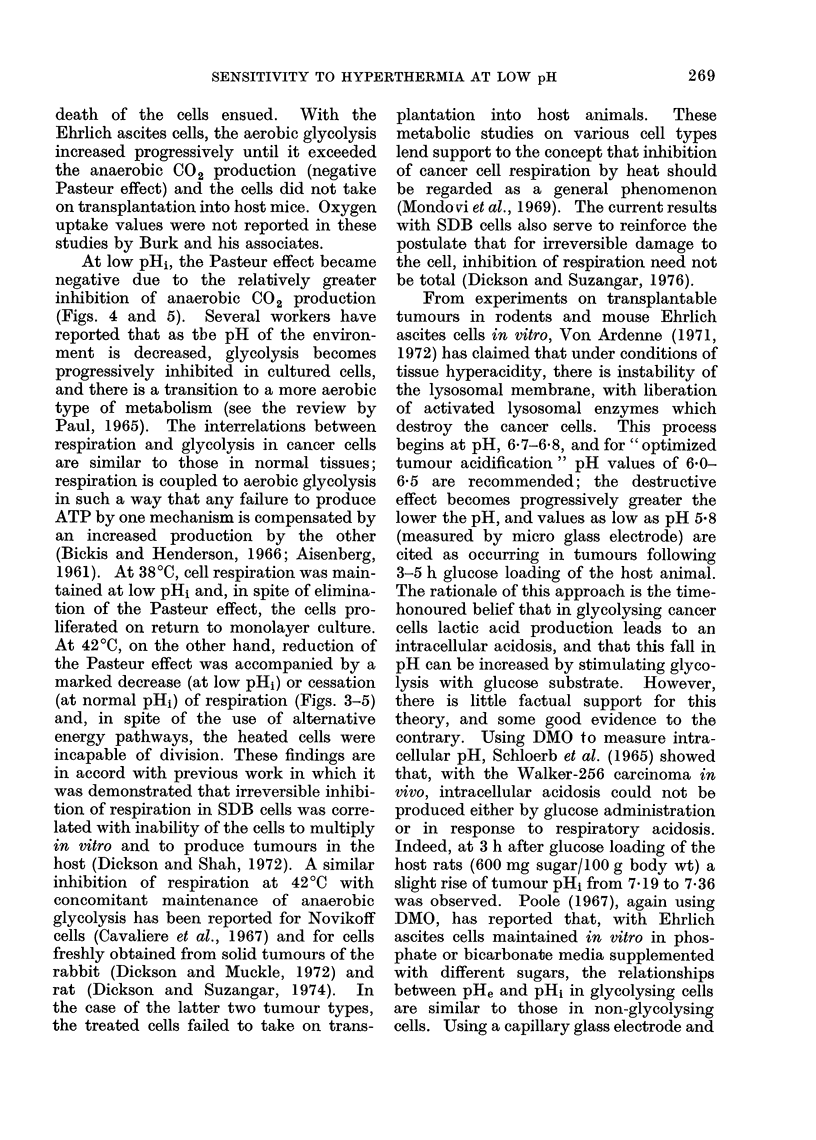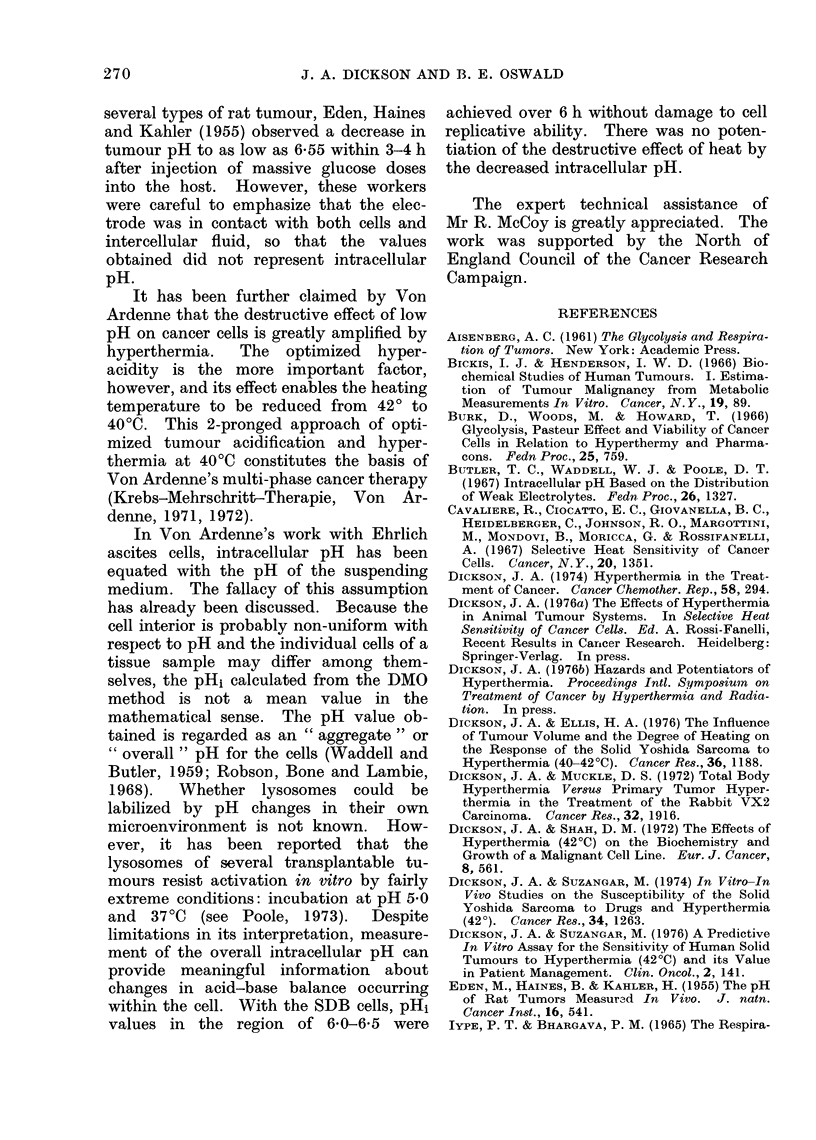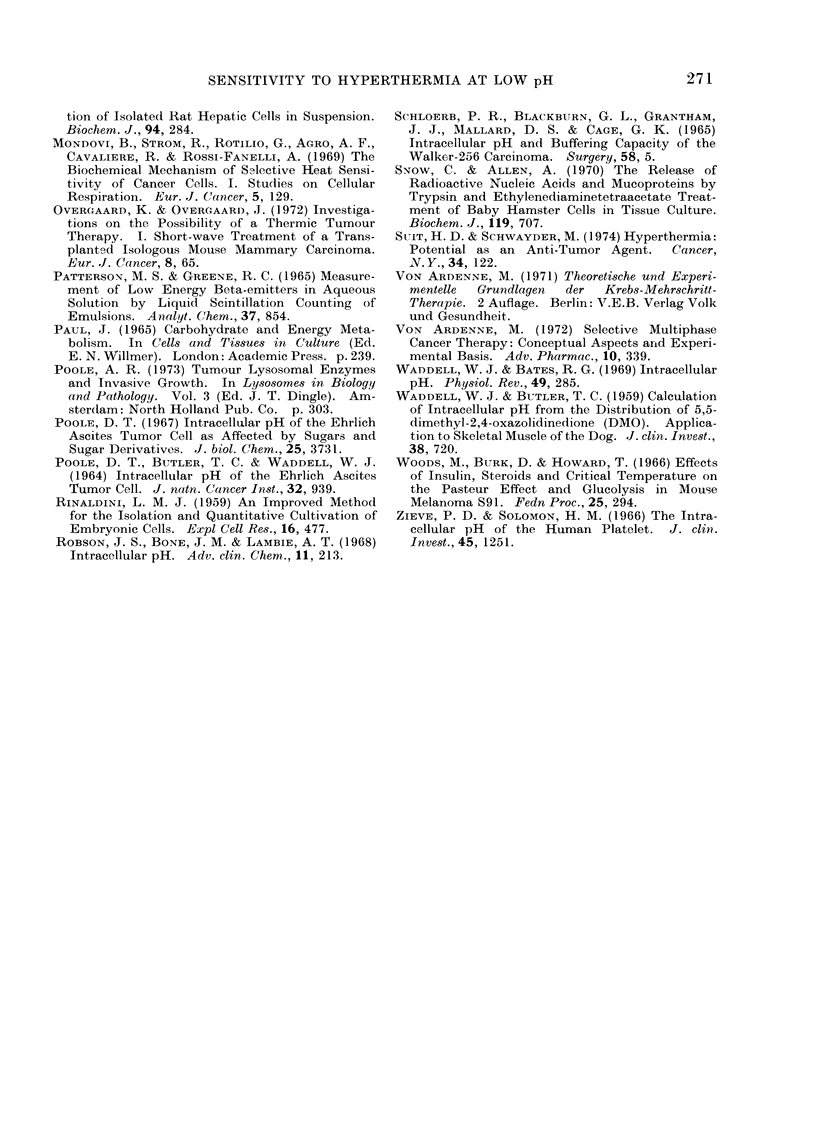# The sensitivity of a malignant cell line to hyperthermia (42 degrees C) at low intracellular pH.

**DOI:** 10.1038/bjc.1976.161

**Published:** 1976-09

**Authors:** J. A. Dickson, B. E. Oswald

## Abstract

The postulate that low intracellular pH acts as a preconditioner for the destructuve effects of hyperthermia (42 degrees C) was examined, using a heat-sensitive line of malignant cells derived from rat mammary gland (SDB). Intracellular pH (pHi) was measured indirectly, from the distribution of the weak, non-metabolizable organic acid 5,5-dimethyl-2,4-oxazolidinedione (DMO) between intra- and extra-cellular water. Respiration, aerobic and anaerobic and anaerobic glycolysis of the cells were studied at normal pHi (pH 7-0-7-4) or at low pHi (pH 6-2-6-6) and at 38 degrees C or 42 degrees C over 6 h in Warburg manometers; the ability of the cells to replicate in culture was examined after 3 h or 6 h incubation in the flasks. The relationship between pHi and extracellular pH (pHe) depended upon the buffer system used and the exact pH in question; no assumption regarding pHi based only on pHe measurement could be made. At 38 degrees C and low pHi, the Pasteur effect became negative due to a relatively greater inhibition of anaerobic than aerobic glycolysis. Respiration was unaffected and cell replicative ability unimpaired. At 42 degrees C and normal pHi, respiration was totally inhibited after 4 h and the Pasteur effect was decreased, in this case due to a compensatory increase in aerobic glycolysis without alteration in anaerobic CO2 production. Low pHi in the presence of hyperthermia enabled cell respiration to continue at a reduced level with no further change in glycolysis. There was delayed cell replication after 3 h at 42 degrees C and inability to multiply following 6 h hyperthermia: low pHi did not influence these results. It is concluded that with these cancer cells, pHi values maintained in the region of 1-0 pH unit below normal for 6 h had no deleterious effect on the cells. No sensitizing effect of the low pHi for the destructive effect of hyperthermia on the cells was observed.


*Br. J. Cancer* (1976) **34**, 262

### THE SENSITIVITY OF A MALIGNANT CELL LINE TO HYPERTHERMIA (42°C) AT LOW INTRACELLULAR pH

J. A. DICKSON AND B. E. OSWALD

*From the Cancer Research Unit, University Department of Clinical Biochemistry, Royal Victoria Infirmary, Newcastle upon Tyne*

Received 7 April 1976 Accepted 25 May 1976

**Summary.—The postulate that low intracellular pH acts as a preconditioner for the destructive effects of hyperthermia (42°C) was examined, using a heat-sensitive line of malignant cells derived from rat mammary gland (SDB). Intracellular pH ($pH_i$) was measured indirectly, from the distribution of the weak, non-metabolizable organic acid 5,5-dimethyl-2,4-oxazolidinedione (DMO) between intra- and extra-cellular water. Respiration, aerobic and anaerobic glycolysis of the cells were studied at normal $pH_i$ (pH 7·0–7·4) or at low $pH_i$ (pH 6·2–6·6) and at 38°C or 42°C over 6 h in Warburg manometers; the ability of the cells to replicate in culture was examined after 3 h or 6 h incubation in the flasks.**

**The relationship between $pH_i$ and extracellular pH ($pH_e$) depended upon the buffer system used and the exact pH in question; no assumption regarding $pH_i$ based only on $pH_e$ measurement could be made. At 38°C and low $pH_i$, the Pasteur effect became negative due to a relatively greater inhibition of anaerobic than aerobic glycolysis. Respiration was unaffected and cell replicative ability unimpaired. At 42°C and normal $pH_i$, respiration was totally inhibited after 4 h and the Pasteur effect was decreased, in this case due to a compensatory increase in aerobic glycolysis without alteration in anaerobic $CO_2$ production. Low $pH_i$ in the presence of hyperthermia enabled cell respiration to continue at a reduced level with no further change in glycolysis. There was delayed cell replication after 3 h at 42°C and inability to multiply following 6 h hyperthermia: low $pH_i$ did not influence these results.**

**It is concluded that with these cancer cells, $pH_i$ values maintained in the region of 1·0 pH unit below normal for 6 h had no deleterious effect on the cells. No sensitizing effect of the low $pH_i$ for the destructive effect of hyperthermia on the cells was observed.**

THE USE of temperatures in the region of 42°C (hyperthermia) to destroy malignant cells is commanding new interest and enthusiasm amongst oncologists. As a therapeutic modality, heat has received intermittent attention since the latter years of the last century with some impressive results in the treatment of animal and human cancer (Cavaliere *et al.*, 1967; Suit and Schwayder, 1974; Dickson and Suzangar, 1976). Before hyperthermia can warrant more widespread clinical application, more information is required on the strategy of heating tumours, and better methods of applying heat are needed, both locally to the tumour and generally to the host, than those now available.

Data currently available indicate that at 42°C there is a selective destructive effect on malignant cells, both *in vitro* and *in vivo* in animal tumours (Cavaliere *et al.*, 1967; Overgaard and Overgaard, 1972; Dickson, 1976*a*) and human cancers (Cavaliere *et al.*, 1967; Dickson and Suzangar, 1976). The difference in heat sensitivity between cancer cells and normal cells decreases with increasing temperature above 42°C (Dickson, 1976*a,b*), so that temperatures in excess of this involve hazard to the host. With heat-sensitive tumours, host cure is dependent upon adequate duration of heating in relation to tumour size (Dickson and Ellis, 1976). With a large tumour burden the prolonged treatment required at 42°C imposes considerable stress on the host (Cavaliere *et al.*, 1967; Dickson, 1974), who may be elderly and in an already debilitated state from his disease. The advantages of sensitizing the cancer cells to the effects of heat are therefore apparent. Several such preconditioners, including methylene blue, dimethylsulphate, tween 80 and glucose, have been proposed by Von Ardenne (1971). Glucose, as the most physiological of these substances, has most appeal. It is claimed that a high level of the sugar in the blood stimulates tumour glycolysis and selectively decreases the pH in the tumour; a difference in the region of 1·0 pH unit between the cancer tissue and normal tissue enables the heating temperature to be reduced to 40°C, and at the same time there is amplification of the damaging effect of the heat on the cancer cells (Von Ardenne, 1971, 1972).

In the present work, the effect of decreased pH on the response of a heat-sensitive line of malignant cells to 42°C has been examined. A measured intracellular pH of approximately 1·0 pH unit below that of normal tissues (taken as pH 7·4) was achieved by maintaining cell populations in buffers of appropriate pH. Metabolism of the cells over 6 h incubation at the elevated temperature, and their subsequent replicative ability at 38°C, were then studied.

### MATERIALS AND METHODS

*Cell line.*—The cell line used was established by one of us (J.A.D.) in 1964. The line was designated " SDB " because it originated from an adenocarcinoma in the breast of a female Sprague–Dawley rat treated with 7,12-dimethylbenzanthracene (Dickson and Shah, 1972). Morphologically, the cells resemble fibroblasts and produce malignant tumours on inoculation into Sprague–Dawley rats and the hosts die within 30 days (Dickson and Shah, 1972). The cells are routinely maintained in 9-cm diameter plastic Petri dishes (Esco, grade AA) on Waymouth medium MB 752/1 containing 10% pooled human AB serum. Cultures are incubated at 38°C in an atmosphere of 5% $CO_2$ in air using a $CO_2$ incubator.

For the present work, cultures in the logarithmic phase of growth (3 days after subculture) were used. For subculture or for experiments, the cells were removed from the Petri dishes with 0·04% crystalline trypsin (Sigma, bovine pancreas type 1, 2 × crystallized) in Rinaldini (1959) saline. This enzyme has been reported to cause release of only 1% of the cellular nucleic acids, with minimal damage to the cell surface, during the harvesting of cultured cells (Snow and Allen, 1970).

*Warburg manometry.*—Respiration and glycolysis were studied by traditional Warburg manometry using an optimal number of cells, 7 to 10 × $10^6$, per flask. For respiration, a Krebs–Ringer phosphate (KRP) solution containing 0·01M sodium succinate was used, with air as the gas phase and 10% KOH in the centre well. Anaerobic glycolysis was measured as $CO_2$ production from a Krebs–Ringer bicarbonate phosphate (KRBP) buffer (Mondovi *et al.*, 1969) containing glucose at 2 g/l. The gas phase was 95% $N_2$/5% $CO_2$ (initial $O_2$ content of mixture less than 20 parts/$10^6$, Air Products Ltd). Aerobic glycolysis was studied by the differential method of Warburg, using two flasks of similar volume with the KRBP buffer and air/5% $CO_2$ as the gas phase. In flask 1, the pressure change (due to $O_2$ consumption, respiratory $CO_2$ production, and $CO_2$ displaced by lactic acid produced from endogenous glycolysis by the cells) served as the zero-point for the alteration in pressure in flask 2, which contained 2 g/l glucose. Previous work with SDB cells indicated that $CO_2$ displacement from bicarbonate buffers equated with lactic acid production by the cells at 38°C and 42°C (Dickson and Shah, 1972). All manometric observations were performed in duplicate flasks, and results were expressed as μl gas exchanged/mg TCA-insoluble dry weight of cells/h (Q value).

*Measurements of pH.*—All buffers for Warburg manometry and for intracellular pH determinations were adjusted for pH at 38°C against standard commercial (BDH) buffers for this temperature. At 42°C, the pH of the buffers was decreased by $< 0·05$ pH units, and consequently the pH of the solutions was not further adjusted for the hyperthermia experiments. A Pye Dynacap pH meter with water-jacketed glass microelectrode was used in preparing the buffers.

For the determination of pH and $pCO_2$ in the experiments on intracellular pH, a Radiometer Microelectrode Unit (PHA 931) and Acid–Base Analyser (PHM 71) with a $pCO_2$ module (PHA 931) were employed; the temperature was maintained at 38°C or 42°C by a Water Thermostat (Radiometer, model VTS 13).

*Intracellular pH determination*
*Principle*

Intracellular pH can be determined indirectly by measurement of the distribution of a weak acid or base between the intracellular and extracellular fluid compartments. The principle of the method is that cell membranes in general behave as if freely permeable to the undissociated forms of weak acids and bases but impermeable to the ionic forms. After introduction of the acid or base into the extracellular compartment, a state of equilibrium is achieved, in which the undissociated form is distributed in equal concentration on either side of the cell membrane. The concentration of the ionized species is then determined solely by the pH and apparent dissociation constant, pK′, of the compound on each side of the membrane, the concentration of the ionic form being directly proportional to the hydrogen ion concentration. The *total* concentration determined analytically, *i.e.* the sum of undissociated and dissociated forms, will be higher on the side on which ionization is more extensive, *viz.* the side of higher pH with an acid, the side of lower pH in the case of a base. Thus, if the total concentration of the indicator compound on both sides of the membrane at equilibrium and the pH on one side of the membrane can be measured, knowing the pK′ of the compound, the pH on the other side of the membrane can be calculated. The indicator concentration is expressed in terms of tissue water, and a correction is made for the compound present in the extracellular fluid. For tissue samples, this requires an estimation of total water content of the sample and of the volume of extracellular fluid present.

The special requirements for a compound to be used as an indicator of intracellular pH exclude the great majority of known acids and bases. In 1959, Waddell and Butler pointed out that the desired attributes were possessed to a high degree by the weak organic acid 5,5-dimethyl-2,4-oxazolidinedione (DMO), and introduced DMO for determination of the intracellular pH of dog muscle. Subsequently, DMO has been used by numerous workers in a variety of intracellular pH investigations, and the reliability and limitations of the acid in this respect have been discussed at length (Waddell and Butler, 1959; Butler, Waddell and Poole, 1967; Waddell and Bates, 1969).

*Methodology*

For the present work, radioactive DMO was employed in conjunction with inulin-carboxyl-$^{14}C$ for measurement of extracellular water, as introduced by Poole, Butler and Waddell (1964). DMO-2-$^{14}C$ (2–10 mCi/mmol) was obtained from New England Nuclear Corporation, and inulin-carboxyl-$^{14}C$ (9·8 mCi/mmol) from The Radiochemical Centre, Amersham.

After harvesting, the SDB cells were washed in buffer of the appropriate pH, counted, and allowed to equilibrate for 30 min in the buffer. The cells were then resuspended in fresh buffer to a final concentration of 25–30% cells. DMO-2-$^{14}C$ (0·05 $\mu$Ci) and inulin-carboxyl-$^{14}C$ (1·0 $\mu$Ci) in 0·1 ml isotonic NaCl were added per ml of cell suspension. Aliquots (usually 1·5–2·0 ml) of the suspension were then pipetted into Warburg flasks and incubated under similar conditions to those used for measuring respiration and glycolysis. The initial pH of the KRP solution was adjusted over the range pH 6·0–8·0 with varying proportions of $Na_2HPO_4$ and $NaH_2PO_4$. With KOH in the centre well, the $pCO_2$ in the flask was negligible. With the KRBP double buffer, pH was varied similarly by altering the phosphate component, the $NaHCO_3$ concentration remaining constant. As in glycolysis experiments, the shaking flasks were continuously flushed for 5 min with 5% $CO_2$ in air to equilibrate the buffer at the beginning of the incubation.

After a 30-min incubation period, a sample of the cell suspension was removed from the flask by means of a long needle and Hamilton syringe. The aliquot was immediately injected into the microelectrode unit of the acid–base analyser. The presence of the cells did not affect the pH or $pCO_2$

determination. Use of the suspension minimized the time available for gas exchange with the atmosphere, and maintained the ratio of cells to supernatant constant for subsequent manipulations. The remaining suspension was removed from the flask and the cells divided equally into two small tared glass-stoppered test tubes (tubes 1 and 2). After centrifugation, the supernatant from each tube was transferred to another pair of tared tubes (tubes 3 and 4). The cell layers were disrupted by repeated freezing and thawing. One tube from each pair was then used for simultaneous determination of DMO-2-$^{14}$C, inulin-carboxyl-$^{14}$C and total cell water.

*Tubes* 1 *and* 3 (for water, inulin and DMO).—The contents of each tube were freeze-dried overnight to constant weight. The desiccated samples were then digested in 2 ml of IN KOH with heating.

*Tubes* 2 *and* 4 (for DMO).—After addition of 1 ml of 5M $NaH_2PO_4$, the tube contents were extracted with 5 ml ethyl acetate-toluene (50/50, v/v). The tube was centrifuged and the upper layer, into which the DMO was confirmed to be quantitatively extracted, was removed.

For radioactive counting, one quarter of the final sample in each tube obtained as described above was transferred to a counting vial and its activity determined using the toluene: Triton X-100 scintillation mixture of Patterson and Greene (1965). The samples were neutralized before addition of the counting fluid, and an internal standard of methanol-$^{14}$C was used to correct for quenching.

*Calculation of intracellular pH.*—Intracellular pH was computed from the equation of Wadell and Butler (1959) expressed in the form proposed by Poole *et al.* (1964) for use with isotopic indicators:

$pH_i = 6{\cdot}13 +$ log of the expression

$$\left\{\left[\frac{Dc}{Ds}\left(\frac{Is}{Is - Ic}\right) - \frac{Ic}{Is - Ic}\right]\left[10^{(pH_e - 6\cdot 13)} + 1\right] - 1\right\}$$

where $6{\cdot}13$ = pK′ for DMO
$pH_i$ = intracellular pH
$pH_e$ = pH of supernatant buffer
Dc = ct/min in DMO cellular layer sample
Ds = ct/min in DMO supernatant sample
Ic = ct/min in inulin cellular layer sample after DMO correction
Is = ct/min in inulin supernatant after DMO correction.

Activity in all cases was expressed as ct/min/mg of water in the sample.

As pointed out by Poole *et al.* (1964), the great convenience of this approach is that DMO and inulin counts enter the equation only as ratios of counts in the cellular layer sample to corresponding counts in the supernatant sample. It is therefore not necessary to know the weights of these compounds added, and the exact amount of either chemical introduced is immaterial.

### RESULTS

### *Intracellular pH in relation to buffer (extracellular) pH*

DMO-2-$^{14}$C reached equilibrium across the SDB cell membrane within 15 min under the various conditions examined. The high glycolytic rate of the cells led to a fall in pH of the KRBP buffer over 30 min. The pH of the buffer at the end of the incubation period was therefore taken as the $pH_e$. The pH of the KRP solution changed little over the experimental period.

In Fig. 1, $pH_i$ is plotted as a function, of $pH_e$ when the cells were incubated in KRP buffers of different pH. A fairly constant $pH_i$ of 7·3–7·5 was maintained in a $pH_e$ ranging from 7·0–8·0. Below a buffer $pH_e$ of 7·0, the $pH_i$ fell progressively with the $pH_e$, but the cell maintained a pH above that of its environment until a $pH_i$ of 6·5 was reached, below which $pH_i$ decreased rapidly.

Fig. 2 indicates that there was a linear relationship between intracellular and extracellular pH values when the cells were incubated in the bicarbonate buffer. Over the $pH_e$ range 6·0–8·0 the cell maintained a slightly higher pH than that of its environment.

A small number of experiments were performed at an incubation temperature of 42°C. With KRP buffers at pH 7·4 and 6·3, and KRBP solutions of pH 7·1 and

FIG. 1.—Relationship of intracellular pH ($pH_i$) to extracellular pH ($pH_e$) of SDB tumour cells incubated in KRP buffer. $pH_e$ was varied by altering the proportions of $Na_2HPO_4$ and $NaH_2PO_4$ in the buffer; $pCO_2$ in the flask was negligible. Each symbol is one determination; the points were usually obtained in pairs, one at low and the other at high $pH_e$.

FIG. 2.—$pH_i$ of SDB tumour cells as a function of $pH_e$ in KRBP buffer. Extracellular pH was varied by adjusting the ratio $Na_2HPO_4/NaH_2PO_4$ in the buffer while maintaining $NaHCO_3$ and $pCO_2$ constant. Results from all determinations are plotted, and the points were usually obtained in pairs as for Fig. 1.

6·3, the calculated $pH_i$ values did not differ significantly from those obtained with similar buffers maintained at 38°C.

*Effect of intracellular pH on cell respiration and glycolysis*

Initial experiments indicated that considerable shifts in $pH_i$ occurred during respiration and glycolysis of SDB cells, especially when the cells were incubated in buffers of low pH (6·2–6·6). In extensive preliminary studies, the suitability of several buffer systems for the present work was considered. For respiration, the chemical buffers imidazole–HCl–sucrose (Mondovi *et al.*, 1969) and Tris–HCl–sucrose (Iype and Bhargava, 1965) in addition to KRP were investigated; for glycolysis, Krebs–Ringer bicarbonate (KRB), KRBP and Tris-HCl–bicarbonate (Dickson and Muckle, 1972) were examined. On the basis of titration curves, $pCO_2$ and percentage $CO_2$ saturation at equilibration (bicarbonate solutions), pH stability and levels of $O_2$ uptake and $CO_2$ production maintained over 6 h with cells in Warburg manometers at 38°C and 42°C and at buffer pH 6·0–8·0, the Krebs–Ringer solutions were selected for use with the SDB cells. The Krebs–Ringer solutions are more physiological in composition than the chemical buffers. In addition, the use of this traditional buffer system maintained the experimental conditions as comparable as possible for studying respiration, aerobic and anaerobic glycolysis.

Figs. 3–5 record gas exchange for SDB populations incubated at normal pH and at low pH. Preliminary experiments revealed that buffer and intracellular pH remained fairly stable for 3 h irrespective of the initial pH of the buffer. Subsequently, shifts of pH occurred, and the $pH_i$ at 6 h was different in the respiration, aerobic and anaerobic flasks. Gas exchange, however, remained linear over the incubation period. The initial pH of the buffers was selected to give a $pH_i$ at 3 h in the range pH 7·2–7·4 (normal pH), or in the range pH 6·3–6·6 (low pH).

FIG. 3.—Oxygen consumption of SDB populations (7–10 × $10^6$ cells) over 6 h in KRP buffer in Warburg manometers with air as the gas phase, and concomitant changes in $pH_i$. Initial $pH_i$ was adjusted by a 30-min preincubation in KRP of appropriate pH, as obtained from Fig. 1. Each point is the mean ± s.d. from 3–4 separate experiments. Solid lines refer to results obtained at incubation temperature of 38°C, broken lines to results at 42°C; solid symbols indicate $pH_i$ in the normal range (values in upper pH record), open symbols denote low $pH_i$ (values in lower pH record).

FIG. 4.—Aerobic $CO_2$ production of SDB populations (7–10 × $10^6$ cells) in Warburg manometers over 6 h, with associated $pH_i$ measurements. The cells were incubated at 38°C or 42°C, and at normal or low $pH_i$, in KRBP with 5% $CO_2$/95% air as the gas phase. Initial $pH_i$ was adjusted by a 30 min preincubation in KRBP, the pH of which was determined from Fig. 2. Otherwise, legend as for Fig. 3.

In KRP buffer of normal pH, intracellular pH varied little over 6 h at 38°C or 42°C (Fig. 3). At 38°C, $O_2$ uptake values at normal and low pH overlapped at each time point to the extent that respiration was clearly not significantly altered by low pH. At 42°C and normal $pH_i$, respiration was approximately halved compared to that at 38°C and was totally inhibited after 4 h. When the $pH_i$ was low, total inhibition of respiration was not observed and the $O_2$ uptake remained linear but reduced throughout the experiment.

Aerobic glycolysis was increased by 50–60% at 42°C when the $pH_i$ remained normal (Fig. 4). At low $pH_i$, aerobic $CO_2$ production was reduced by 45–60% and was similar at 38°C and 42°C.

Fig. 5 shows that at normal $pH_i$ the rate of anaerobic glycolysis of the cells was approximately twice the rate of aerobic glycolysis, and was unaffected by elevated temperature. When the intracellular pH was low, anaerobic $CO_2$ production was reduced to 30% of its value at normal pH. Again, there was no significant difference between glycolysis at 38°C and 42°C.

*Viability of incubated cells*

After incubation periods of 3 h or 6 h, the cells were removed from the Warburg flasks, their viability assessed using trypan blue dye, and they were then resuspended in culture medium at 38°C and transferred to Petri dishes. Cells incubated at 38°C and normal or low $pH_i$ for 3 h had a viability of 90–95%, and entered logarithmic growth after 24 h in culture. After 3 h at 42°C, 40–50% of the cells removed from respiration or glycolysis flasks stained

FIG. 5.—Anaerobic $CO_2$ production of SDB populations (7–10 × $10^6$ cells) over 6 h at 38°C or 42°C, and at normal or low $pH_i$, with associated $pH_i$ profiles. The cells were incubated in KRBP buffer with a gas phase of 5% $CO_2$/95% $N_2$. Initial $pH_i$ was adjusted by a 30-min preincubation in KRBP, the pH of which was obtained from Fig. 2. Otherwise, legend as for Fig. 3.

with trypan blue. Proliferation occurred in Petri dishes and the cells entered the log phase of growth after 3 days. There was no difference in morphology or behaviour between cells that had been incubated at normal $pH_i$ or at low $pH_i$. At the end of 6 h, the Warburg populations maintained at 38°C were 85–90% viable, and on culture the cells were multiplying exponentially within 36 h. In populations subjected to 42°C for 6 h, less than 10% of the cells remained unstained by trypan blue, and no proliferation occurred in culture. Cells incubated at normal or low $pH_i$ behaved similarly.

DISCUSSION

In the present experiments, intracellular pH was maintained at a level higher than the extracellular pH in the buffers used, the precise relationship between $pH_i$ and $pH_e$ depending on the buffer and the pH in question (Figs. 1 and 2). Work *in vitro* with Ehrlich ascites cells (Poole *et al.*, 1964) and human platelets (Zieve and Solomon, 1966) has shown that the relationship between $pH_i$ and $pH_e$ may not remain constant over the range pH 6·0–8·0. Indeed, with glycolysing Ehrlich ascites cells in bicarbonate buffer, the $pH_i$ may be rising at a time when the $pH_e$ is falling (Poole *et al.*, 1964). The SDB results, therefore, reemphasize the conclusion of both these groups of earlier workers that no inference as to intracellular pH based only on knowledge of extracellular pH can be made with any assurance.

The SDB cells exhibited a marked Pasteur effect (inhibition of glycolysis by oxygen). The most meaningful expression of this effect is $Q_{CO_2}^{N_2}$–$Q_{CO_2}^{O_2}$ (the absolute Pasteur effect (Aisenberg, 1961)). In a gas phase of 95% $O_2$/5% $CO_2$ aerobic glycolysis values for the cells were not significantly different to the Q values obtained with 95% air/5% $CO_2$. Computed on this basis, the absolute Pasteur effect of the SDB cells was 20 at 38°C, a value in the lower range of magnitude for the effect as found in pure populations of cancer cells, *e.g.* Krebs ascites cells (Aisenberg, 1961). At 42°C, inhibition of $O_2$ uptake by the cells (Fig. 3) was accompanied by a marked increase in aerobic glycolysis (Fig. 4) and little change in anaerobic glycolysis (Fig. 5), with a consequent decrease in the Pasteur effect to a value of 5. Burk and his group reported elimination of the Pasteur effect by increased aerobic glycolysis in mouse melanoma S91 cells at 43°C (Woods, Burk and Howard, 1966) and in Ehrlich ascites cells at 45°C (Burk, Woods and Howard, 1966). These workers regard this elimination of the Pasteur effect as a " metabolic uncoupling " which is unfavourable to the malignant cells. In the case of the melanoma cells, following uncoupling, aerobic glycolysis decayed and metabolic death of the cells ensued. With the Ehrlich ascites cells, the aerobic glycolysis increased progressively until it exceeded the anaerobic $CO_2$ production (negative Pasteur effect) and the cells did not take on transplantation into host mice. Oxygen uptake values were not reported in these studies by Burk and his associates.

At low $pH_i$, the Pasteur effect became negative due to the relatively greater inhibition of anaerobic $CO_2$ production (Figs. 4 and 5). Several workers have reported that as tbe pH of the environment is decreased, glycolysis becomes progressively inhibited in cultured cells, and there is a transition to a more aerobic type of metabolism (see the review by Paul, 1965). The interrelations between respiration and glycolysis in cancer cells are similar to those in normal tissues; respiration is coupled to aerobic glycolysis in such a way that any failure to produce ATP by one mechanism is compensated by an increased production by the other (Bickis and Henderson, 1966; Aisenberg, 1961). At 38°C, cell respiration was maintained at low $pH_i$ and, in spite of elimination of the Pasteur effect, the cells proliferated on return to monolayer culture. At 42°C, on the other hand, reduction of the Pasteur effect was accompanied by a marked decrease (at low $pH_i$) or cessation (at normal $pH_i$) of respiration (Figs. 3–5) and, in spite of the use of alternative energy pathways, the heated cells were incapable of division. These findings are in accord with previous work in which it was demonstrated that irreversible inhibition of respiration in SDB cells was correlated with inability of the cells to multiply *in vitro* and to produce tumours in the host (Dickson and Shah, 1972). A similar inhibition of respiration at 42°C with concomitant maintenance of anaerobic glycolysis has been reported for Novikoff cells (Cavaliere *et al.*, 1967) and for cells freshly obtained from solid tumours of the rabbit (Dickson and Muckle, 1972) and rat (Dickson and Suzangar, 1974). In the case of the latter two tumour types, the treated cells failed to take on transplantation into host animals. These metabolic studies on various cell types lend support to the concept that inhibition of cancer cell respiration by heat should be regarded as a general phenomenon (Mondovi *et al.*, 1969). The current results with SDB cells also serve to reinforce the postulate that for irreversible damage to the cell, inhibition of respiration need not be total (Dickson and Suzangar, 1976).

From experiments on transplantable tumours in rodents and mouse Ehrlich ascites cells *in vitro*, Von Ardenne (1971, 1972) has claimed that under conditions of tissue hyperacidity, there is instability of the lysosomal membrane, with liberation of activated lysosomal enzymes which destroy the cancer cells. This process begins at pH, 6·7–6·8, and for "optimized tumour acidification" pH values of 6·0–6·5 are recommended; the destructive effect becomes progressively greater the lower the pH, and values as low as pH 5·8 (measured by micro glass electrode) are cited as occurring in tumours following 3–5 h glucose loading of the host animal. The rationale of this approach is the time-honoured belief that in glycolysing cancer cells lactic acid production leads to an intracellular acidosis, and that this fall in pH can be increased by stimulating glycolysis with glucose substrate. However, there is little factual support for this theory, and some good evidence to the contrary. Using DMO to measure intracellular pH, Schloerb *et al.* (1965) showed that, with the Walker-256 carcinoma *in vivo*, intracellular acidosis could not be produced either by glucose administration or in response to respiratory acidosis. Indeed, at 3 h after glucose loading of the host rats (600 mg sugar/100 g body wt) a slight rise of tumour $pH_i$ from 7·19 to 7·36 was observed. Poole (1967), again using DMO, has reported that, with Ehrlich ascites cells maintained *in vitro* in phosphate or bicarbonate media supplemented with different sugars, the relationships between $pH_e$ and $pH_i$ in glycolysing cells are similar to those in non-glycolysing cells. Using a capillary glass electrode and several types of rat tumour, Eden, Haines and Kahler (1955) observed a decrease in tumour pH to as low as 6·55 within 3–4 h after injection of massive glucose doses into the host. However, these workers were careful to emphasize that the electrode was in contact with both cells and intercellular fluid, so that the values obtained did not represent intracellular pH.

It has been further claimed by Von Ardenne that the destructive effect of low pH on cancer cells is greatly amplified by hyperthermia. The optimized hyperacidity is the more important factor, however, and its effect enables the heating temperature to be reduced from 42° to 40°C. This 2-pronged approach of optimized tumour acidification and hyperthermia at 40°C constitutes the basis of Von Ardenne's multi-phase cancer therapy (Krebs–Mehrschritt–Therapie, Von Ardenne, 1971, 1972).

In Von Ardenne's work with Ehrlich ascites cells, intracellular pH has been equated with the pH of the suspending medium. The fallacy of this assumption has already been discussed. Because the cell interior is probably non-uniform with respect to pH and the individual cells of a tissue sample may differ among themselves, the $pH_i$ calculated from the DMO method is not a mean value in the mathematical sense. The pH value obtained is regarded as an " aggregate " or " overall " pH for the cells (Waddell and Butler, 1959; Robson, Bone and Lambie, 1968). Whether lysosomes could be labilized by pH changes in their own microenvironment is not known. However, it has been reported that the lysosomes of several transplantable tumours resist activation *in vitro* by fairly extreme conditions: incubation at pH 5·0 and 37°C (see Poole, 1973). Despite limitations in its interpretation, measurement of the overall intracellular pH can provide meaningful information about changes in acid–base balance occurring within the cell. With the SDB cells, $pH_i$ values in the region of 6·0–6·5 were achieved over 6 h without damage to cell replicative ability. There was no potentiation of the destructive effect of heat by the decreased intracellular pH.

The expert technical assistance of Mr R. McCoy is greatly appreciated. The work was supported by the North of England Council of the Cancer Research Campaign.

### REFERENCES

AISENBERG, A. C. (1961) *The Glycolysis and Respiration of Tumors.* New York: Academic Press.

BICKIS, I. J. & HENDERSON, I. W. D. (1966) Biochemical Studies of Human Tumours. I. Estimation of Tumour Malignancy from Metabolic Measurements *In Vitro. Cancer, N.Y.*, **19**, 89.

BURK, D., WOODS, M. & HOWARD, T. (1966) Glycolysis, Pasteur Effect and Viability of Cancer Cells in Relation to Hyperthermy and Pharmacons. *Fedn Proc.*, **25**, 759.

BUTLER, T. C., WADDELL, W. J. & POOLE, D. T. (1967) Intracellular pH Based on the Distribution of Weak Electrolytes. *Fedn Proc.*, **26**, 1327.

CAVALIERE, R., CIOCATTO, E. C., GIOVANELLA, B. C., HEIDELBERGER, C., JOHNSON, R. O., MARGOTTINI, M., MONDOVI, B., MORICCA, G. & ROSSIFANELLI, A. (1967) Selective Heat Sensitivity of Cancer Cells. *Cancer, N.Y.*, **20**, 1351.

DICKSON, J. A. (1974) Hyperthermia in the Treatment of Cancer. *Cancer Chemother. Rep.*, **58**, 294.

DICKSON, J. A. (1976*a*) The Effects of Hyperthermia in Animal Tumour Systems. In *Selective Heat Sensitivity of Cancer Cells. Ed.* A. Rossi-Fanelli, Recent Results in Cancer Research. Heidelberg: Springer-Verlag. In press.

DICKSON, J. A. (1976*b*) Hazards and Potentiators of Hyperthermia. *Proceedings Intl. Symposium on Treatment of Cancer by Hyperthermia and Radiation.* In press.

DICKSON, J. A. & ELLIS, H. A. (1976) The Influence of Tumour Volume and the Degree of Heating on the Response of the Solid Yoshida Sarcoma to Hyperthermia (40–42°C). *Cancer Res.*, **36**, 1188.

DICKSON, J. A. & MUCKLE, D. S. (1972) Total Body Hyperthermia *Versus* Primary Tumor Hyperthermia in the Treatment of the Rabbit VX2 Carcinoma. *Cancer Res.*, **32**, 1916.

DICKSON, J. A. & SHAH, D. M. (1972) The Effects of Hyperthermia (42°C) on the Biochemistry and Growth of a Malignant Cell Line. *Eur. J. Cancer*, **8**, 561.

DICKSON, J. A. & SUZANGAR, M. (1974) *In Vitro–In Vivo* Studies on the Susceptibility of the Solid Yoshida Sarcoma to Drugs and Hyperthermia (42°). *Cancer Res.*, **34**, 1263.

DICKSON, J. A. & SUZANGAR, M. (1976) A Predictive *In Vitro* Assay for the Sensitivity of Human Solid Tumours to Hyperthermia (42°C) and its Value in Patient Management. *Clin. Oncol.*, **2**, 141.

EDEN, M., HAINES, B. & KAHLER, H. (1955) The pH of Rat Tumors Measured *In Vivo. J. natn. Cancer Inst.*, **16**, 541.

IYPE, P. T. & BHARGAVA, P. M. (1965) The Respiration of Isolated Rat Hepatic Cells in Suspension. *Biochem. J.*, **94**, 284.
MONDOVI, B., STROM, R., ROTILIO, G., AGRO, A. F., CAVALIERE, R. & ROSSI-FANELLI, A. (1969) The Biochemical Mechanism of Selective Heat Sensitivity of Cancer Cells. I. Studies on Cellular Respiration. *Eur. J. Cancer*, **5**, 129.
OVERGAARD, K. & OVERGAARD, J. (1972) Investigations on the Possibility of a Thermic Tumour Therapy. I. Short-wave Treatment of a Transplanted Isologous Mouse Mammary Carcinoma. *Eur. J. Cancer*, **8**, 65.
PATTERSON, M. S. & GREENE, R. C. (1965) Measurement of Low Energy Beta-emitters in Aqueous Solution by Liquid Scintillation Counting of Emulsions. *Analyt. Chem.*, **37**, 854.
PAUL, J. (1965) Carbohydrate and Energy Metabolism. In *Cells and Tissues in Culture* (Ed. E. N. Willmer). London: Academic Press. p. 239.
POOLE, A. R. (1973) Tumour Lysosomal Enzymes and Invasive Growth. In *Lysosomes in Biology and Pathology*. Vol. 3 (Ed. J. T. Dingle). Amsterdam: North Holland Pub. Co. p. 303.
POOLE, D. T. (1967) Intracellular pH of the Ehrlich Ascites Tumor Cell as Affected by Sugars and Sugar Derivatives. *J. biol. Chem.*, **25**, 3731.
POOLE, D. T., BUTLER, T. C. & WADDELL, W. J. (1964) Intracellular pH of the Ehrlich Ascites Tumor Cell. *J. natn. Cancer Inst.*, **32**, 939.
RINALDINI, L. M. J. (1959) An Improved Method for the Isolation and Quantitative Cultivation of Embryonic Cells. *Expl Cell Res.*, **16**, 477.
ROBSON, J. S., BONE, J. M. & LAMBIE, A. T. (1968) Intracellular pH. *Adv. clin. Chem.*, **11**, 213.
SCHLOERB, P. R., BLACKBURN, G. L., GRANTHAM, J. J., MALLARD, D. S. & CAGE, G. K. (1965) Intracellular pH and Buffering Capacity of the Walker-256 Carcinoma. *Surgery*, **58**, 5.
SNOW, C. & ALLEN, A. (1970) The Release of Radioactive Nucleic Acids and Mucoproteins by Trypsin and Ethylenediaminetetraacetate Treatment of Baby Hamster Cells in Tissue Culture. *Biochem. J.*, **119**, 707.
SUIT, H. D. & SCHWAYDER, M. (1974) Hyperthermia: Potential as an Anti-Tumor Agent. *Cancer, N.Y.*, **34**, 122.
VON ARDENNE, M. (1971) *Theoretische und Experimentelle Grundlagen der Krebs-Mehrschritt-Therapie*. 2 Auflage. Berlin: V.E.B. Verlag Volk und Gesundheit.
VON ARDENNE, M. (1972) Selective Multiphase Cancer Therapy: Conceptual Aspects and Experimental Basis. *Adv. Pharmac.*, **10**, 339.
WADDELL, W. J. & BATES, R. G. (1969) Intracellular pH. *Physiol. Rev.*, **49**, 285.
WADDELL, W. J. & BUTLER, T. C. (1959) Calculation of Intracellular pH from the Distribution of 5,5-dimethyl-2,4-oxazolidinedione (DMO). Application to Skeletal Muscle of the Dog. *J. clin. Invest.*, **38**, 720.
WOODS, M., BURK, D. & HOWARD, T. (1966) Effects of Insulin, Steroids and Critical Temperature on the Pasteur Effect and Glucolysis in Mouse Melanoma S91. *Fedn Proc.*, **25**, 294.
ZIEVE, P. D. & SOLOMON, H. M. (1966) The Intracellular pH of the Human Platelet. *J. clin. Invest.*, **45**, 1251.